# A Computational Analysis of Alternative Splicing across Mammalian Tissues Reveals Circadian and Ultradian Rhythms in Splicing Events

**DOI:** 10.3390/ijms20163977

**Published:** 2019-08-15

**Authors:** Rukeia El-Athman, Dora Knezevic, Luise Fuhr, Angela Relógio

**Affiliations:** 1 Institute for Theoretical Biology (ITB), Charité-Universitätsmedizin Berlin, corporate member of Freie Universität Berlin, Humboldt-Universität zu Berlin and Berlin Institute of Health, 10117 Berlin, Germany; 2 Medical Department of Hematology, Oncology and Tumor Immunology, and Molekulares Krebsforschungszentrum (MKFZ), Charité-Universitätsmedizin Berlin, corporate member of Freie Universität Berlin, Humboldt-Universität zu Berlin, and Berlin Institute of Health, 10117 Berlin, Germany

**Keywords:** circadian rhythms, ultradian rhythms, alternative splicing, splicing factors, splice isoforms, cancer, tumour progression

## Abstract

Mounting evidence points to a role of the circadian clock in the temporal regulation of post-transcriptional processes in mammals, including alternative splicing (AS). In this study, we carried out a computational analysis of circadian and ultradian rhythms on the transcriptome level to characterise the landscape of rhythmic AS events in published datasets covering 76 tissues from mouse and olive baboon. Splicing-related genes with 24-h rhythmic expression patterns showed a bimodal distribution of peak phases across tissues and species, indicating that they might be controlled by the circadian clock. On the output level, we identified putative oscillating AS events in murine microarray data and pairs of differentially rhythmic splice isoforms of the same gene in baboon RNA-seq data that peaked at opposing times of the day and included oncogenes and tumour suppressors. We further explored these findings using a new circadian RNA-seq dataset of human colorectal cancer cell lines. Rhythmic isoform expression patterns differed between the primary tumour and the metastatic cell line and were associated with cancer-related biological processes, indicating a functional role of rhythmic AS that might be implicated in tumour progression. Our data shows that rhythmic AS events are widespread across mammalian tissues and might contribute to a temporal diversification of the proteome.

## 1. Introduction

Alternative splicing (AS) describes the differential inclusion or exclusion of pre-mRNA regions into protein-coding mRNA that affects approximately 95% of human multi-exon genes [1,2]. The production of multiple protein isoforms with related, distinct and sometimes even antagonistic properties from a single gene is a critical determinant of proteome diversification both on the spatial level, e.g., by contributing to cell type and tissue specificity, and on the temporal level, e.g., in different developmental stages [3]. Aberrant AS is associated with various human pathologies, including cancer, where splicing switches are linked to several cancer hallmarks [4,5,6,7]. It has been suggested that the precise timing of splicing is important for the dynamic control of AS decisions [8]. Moreover, 24-h transcriptional rhythms of splicing-related genes and AS events have been identified in mammalian cells both in vivo [9] and in vitro [10,11] and Titin isoform expression changes have been observed in the muscles of mice with a skeletal muscle-specific knockout of the core circadian clock gene *Arntl* [12]. These findings suggest a circadian control of the splicing process [13] which might result in the time-dependent production of functionally distinct protein isoforms of the same gene.

Cellular timing is regulated by the circadian clock, a self-sustained endogenous oscillator driven by transcriptional and translational feedback loops that generate tissue-specific ~24-h rhythms in the expression of clock-controlled genes and proteins [14]. Circadian rhythms in transcription and—to a lesser extent—their higher-order harmonics, also known as ultradian rhythms, are prevalent across mammalian tissues and cell lines [15,16,17,18,19]. However, so far, high-throughput transcriptome circadian studies usually analyse oscillations on whole gene-level and do not consider the rhythmic expression of individual splice isoforms, thus missing a potential additional layer of circadian post-transcriptional regulation.

Here, we present a systematic genome-wide evaluation of circadian and ultradian rhythms in transcription in the two most comprehensive circadian datasets of mammalian tissues available to date: a microarray dataset of twelve mouse organs [15] and a recently published RNA-sequencing (RNA-seq) dataset of 64 tissues and brain regions from the olive baboon [16]. Our analysis revealed splicing-related genes with consistently circadian expression patterns across tissues and species in mouse and baboon, as well as 24-h and 12-h rhythmic putative AS events in the murine microarray data. We further developed a workflow for the identification and quantification of rhythmic isoform changes in time-course RNA-seq data by comparing transcript-level oscillations of splice isoforms encoded by the same gene. We identified pairs of differentially 24-h rhythmic isoforms in baboon tissues whose expression patterns were shifted by several hours and peaked at opposing times of the daily cycle. Functional annotation of candidate genes with phase-shifted isoforms suggests a reciprocal interplay between circadian AS and the splicing process itself and revealed several oncogenes and tumour suppressors among the candidate genes with rhythmic AS events in multiple baboon tissues. To further explore these findings, we complemented our study with a novel RNA-seq dataset of two human colorectal cancer (CRC) cell lines, SW480 and SW620, that were derived from the same patient from a primary tumour and a metastasis, respectively, and that are known to display distinct circadian phenotypes [10,20]. We identified differing sets of phase-shifted transcript pairs in both cell lines, some of which were implicated in cancer-related biological processes. Overall, our findings point to an additional temporal layer of proteome diversification in mammals via rhythmic AS that results in the production of distinct splice isoforms expressed at different times and may account for a time-dependent function of the resulting proteins.

## 2. Results

### 2.1. Rhythmicity Analysis of Mammalian Tissues Reveals Differences between 24-h and 12-h Rhythmic Features at the Gene- and at the Transcript-Level

**Figure 1:**
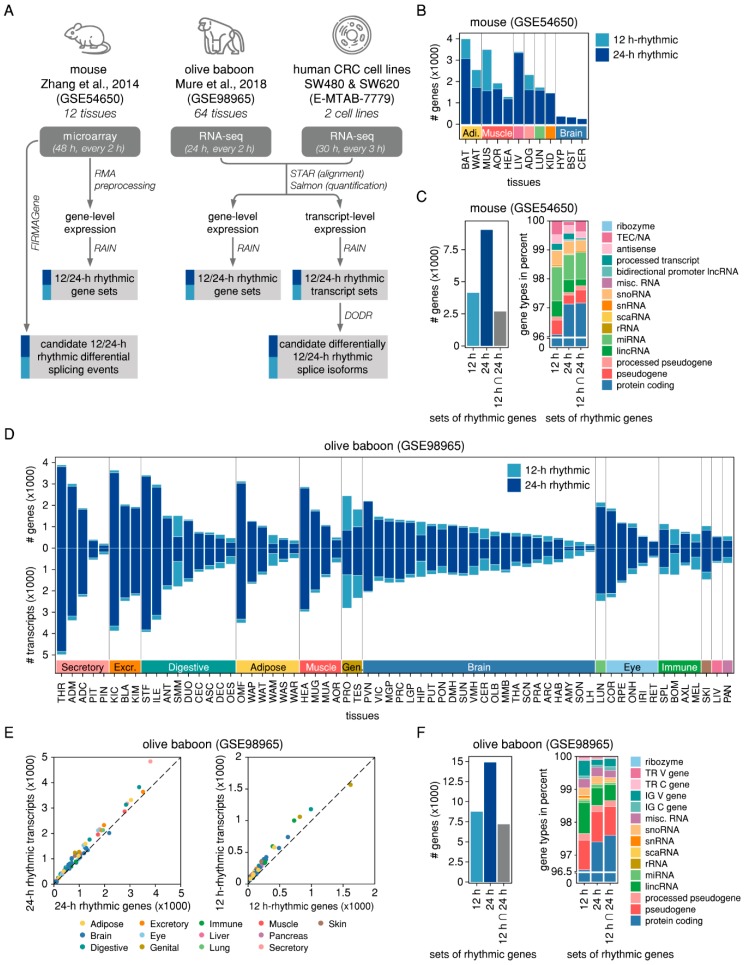
Identification of 24-h and 12-h rhythmic features in nocturnal and diurnal mammalian tissues. (**A**) Workflow of the analysis. The input data consists of three mammalian circadian transcriptome datasets: a microarray dataset of twelve murine tissues (GSE54650), an RNA-seq dataset of 64 olive baboon tissues (GSE98965), and a novel RNA-seq dataset of two human CRC cell lines (E-MTAB-7779). For the microarray data, gene-level expression values were calculated based on RMA-preprocessed intensity values. For the RNA-seq data, sequencing reads were aligned to the transcriptome and both transcript-level and summarised gene-level abundances were estimated. 12-h and 24-h rhythmic gene and transcript sets were identified using RAIN. Candidate AS events in the microarray data were identified using FIRMAGene and candidate differentially rhythmic isoforms in the RNA-seq data were identified using DODR. (**B**) Number of 12-h (light blue) and 24-h rhythmic (dark blue) genes in twelve murine tissues. (**C**) Number of genes (left panel) and gene types in percent (right panel) for the total sets of 12-h and 24-h rhythmic genes across all murine tissues and their intersection. (**E**) Number of 12-h (light blue) and 24-h rhythmic (dark blue) genes (upper panel) and transcripts (lower panel) in 64 baboon tissues. Each of the baboon tissue samples is represented according to the number of 24-h rhythmic transcripts (*y*-axis) and the number of 24-h rhythmic genes (*x*-axis) (left panel) and to the number of 12-h rhythmic transcripts (*y*-axis) and the number of 12-h rhythmic genes (*x*-axis) (right panel). (**F**) Number of genes (left panel) and gene types in percent (right panel) for the total sets of 12-h and 24-h rhythmic genes across all baboon tissues and their intersection.

Circadian rhythms in transcription are ubiquitous in mammalian cells. To investigate whether transcriptomic rhythmicity differs when analysed at the transcript rather than at the whole gene-level, we identified oscillations of both transcripts and genes in an RNA-seq dataset of 64 tissues from the olive baboon [16] and compared them to oscillating genes identified in a microarray dataset of twelve mouse organs [15] (Figure 1A, Table S6). Both datasets include samples taken at discrete circadian time points (every 2 h) for two circadian cycles (48 h) in the mouse and one circadian cycle (24 h) in the baboon, resulting in a total of 1055 samples. While the mice were kept under a constant darkness (DD) cycle after having been entrained to an 12 h light: 12 h dark (LD) cycle for a week) with sampling starting 18 h post release into darkness, the baboons were kept under an LD cycle.

A harmonic regression analysis revealed clusters of rhythmic genes for periods of circadian length (~24 h) and for ultradian periods at the second harmonic of circadian rhythmicity (~12 h) (Figure S1). For the mouse, we identified 9045 genes as 24-h rhythmic and 4140 genes as 12-h rhythmic (RAIN *q* < 0.05 after pre-filtering of RAIN *p*-values for genes with relative amplitude ≥ 0.1) in at least one tissue (Figure 1B). 2673 genes were detected as both 24-h and 12-h rhythmic, depending on the tissue (Figure 1C). In the baboon dataset, we identified 14,881 genes as 24-h rhythmic and 8763 genes as 12-h rhythmic (RAIN *p* < 0.005 and relative amplitude ≥ 0.1) in at least one tissue (Figure 1D,F). In agreement with the observation for the murine tissues, most rhythmic baboon genes (82%) showed oscillations with periods of both 24-h and 12-h, depending on the tissue (Figure 1F). Applying the same criteria for the transcript-level counts, 21,379 transcripts were identified as 24-h rhythmic and 12,941 transcripts as 12-h rhythmic in baboon tissues (Figure 1D), resulting in a roughly linear correlation between the number of rhythmic genes and the number of rhythmic transcripts (Figure 1E).

**Figure 2:**
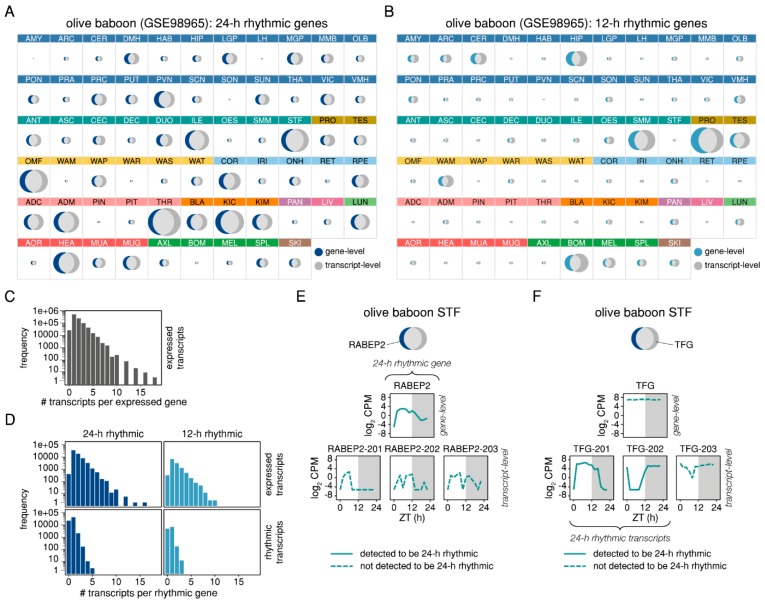
Differences between the sets of genes identified as rhythmic on gene- and on transcript-level in baboon tissues. Tissue-wise area-proportional Venn diagrams of the sets of genes that were identified as (**A**) 24-h rhythmic and (**B**) 12-h rhythmic at gene-level (24-h rhythmic: dark blue; 12-h rhythmic: light blue) and at transcript-level (dark grey) for the baboon data. The intersection of the two sets is represented in light grey. (**C**) Frequency barplot of the number of expressed transcripts per expressed genes in all baboon tissues. (**D**) Frequency barplots of the number of expressed transcripts per 24-h rhythmic gene (dark blue, upper left panel) and per 12-h rhythmic gene (light blue, upper right panel), and of the number of 24-h rhythmic transcripts per 24-h rhythmic gene (dark blue, lower left panel) and of 12-h rhythmic transcripts per 12-h rhythmic gene (light blue, lower right panel) in all baboon tissues. (**E**) Example of a gene (*RABEP2*) that was detected to be 24-h rhythmic in baboon stomach fundus (STF) at gene-level but not at transcript-level. (**F**) Example of a gene (*TFG*) that was detected to be 24-h rhythmic in baboon STF at transcript-level but not at gene-level.

When comparing the identity of the rhythmic genes identified either at the gene or at the transcript-level for each baboon tissue, we observed that on average, the number of genes in the intersection of the two gene sets was only 40% of the set union for the 24-h rhythmic genes and only 25% of the set union for the 12-h rhythmic genes (Figure 2A,B). Interestingly, the tissue-wise median of RAIN *p*-values of genes from the intersection (identified as rhythmic at both the gene and transcript-level) was generally lower than that of the genes identified as rhythmic at gene-level only (Figure S2A). While the phase distributions of rhythmic features in individual tissues were similar between both the gene and the transcript-level (Figure S2B), both 24-h and 12-h rhythmic transcript sets tended to have higher relative amplitudes than their counterparts at gene-level (Figure S2C). While across all baboon tissues, an expressed gene had on average 1.7 expressed transcripts, some genes exhibited a significantly higher number of expressed transcripts, the maximum being 18 for *EPB41L2*, *RACK1*, and *ENSPANG00000011737*, a novel orthologous gene to murine gene *Dhrs4* (Figure 2C). A similar ratio was observed for the average number of expressed transcripts per rhythmic gene (24-h rhythmic: 1.80; 12-h rhythmic: 1.68), while the average number of rhythmic transcripts per rhythmic gene was lower (24-h rhythmic: 0.71; 12-h rhythmic: 0.60), the maximum being five 24-h rhythmic transcripts for *MT3* and *RACK1* (Figure 2D). For genes with several expressed transcripts, we further observed a discrepancy between the expression pattern of the gene and the expression patterns of its individual transcripts. For instance, the gene *RABEP2* was identified as 24-h rhythmic in baboon stomach fundus (STF) but its three expressed transcripts were not (Figure 2E). In contrast, two of the three expressed transcripts of the gene *TFG* were identified as 24-h rhythmic in STF, albeit with different phases, while the summarised expression of *TFG* at gene-level was arrhythmic (Figure 2F). In the first case, the summarisation of transcript-level estimates to gene-level led to a gene being defined as rhythmic despite the individual transcripts being arrhythmic, while in the second case, it led to a masking of the (phase-shifted) rhythmicity of the individual transcripts. Hence, the rhythmicity analysis at transcript-level is likely to yield new results that would be overlooked at gene-level.

### 2.2. Splicing-Related Genes with 24-h Rhythmic Expression Patterns are Conserved Across Mouse and Baboon Tissues and Show Daily and Nightly Clusters of Peak Expression

**Figure 3:**
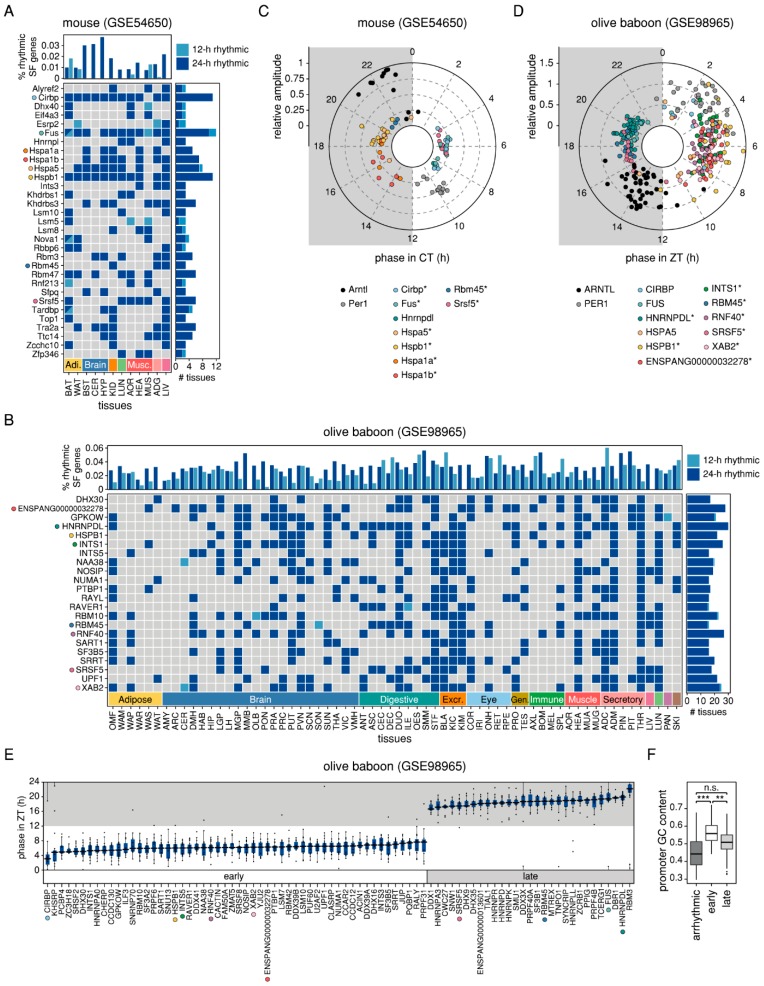
Consistent 24-h rhythmicity of splicing-related genes in mouse and baboon tissues. Categorical heatmap for splicing-related genes that were detected as rhythmic (24-h rhythmic: dark blue; 12-h rhythmic: light blue) in at least three of the murine tissues (**A**) and in at least 16 of the baboon tissues (**B**). The respective upper panels represent the percentage of all rhythmic splicing-related genes in the set of all rhythmic genes per tissue and the respective right panels represent the number of tissues where the splicing-related genes were detected as rhythmic. Distribution of peak phases of selected consistently 24-h rhythmic splicing-related genes and the core-clock components *Arntl*/*ARNTL* and *Per1*/*PER1* in the tissues from mouse (**C**) and baboon (**D**) in which they were detected as 24-h rhythmic. Genes found to be consistently splicing in the respective organism are marked with an asterisk. (**E**) Circular boxplots of peak phases of splicing-related genes that were detected as 24-h rhythmic in at least ten baboon tissues. (**F**) Promoter GC content in sets of arrhythmic, early peaking and late-peaking 24-h rhythmic splicing-related genes calculated for TSS ± 1,000 bp. Wilcoxon rank sum was used to check for significant differences (n.s.: not significant, **: *p* < 0.01 ***: *p* < 0.001). Boxplots show (circular) median and inter-quartile range (IQR). IQR is extended with whiskers to the largest and smallest value, respectively, but no further than 1.5 × IQR from hinges.

The differential expression of multiple isoforms of the same gene can be attributed to AS of its pre-mRNA, a process that is catalysed by the spliceosome and regulated by auxiliary splicing factors [21]. We examined the transcriptional expression and rhythmic parameters of 404 human splicing-related genes that were mapped to 451 orthologous murine genes and to 429 baboon genes of which 409 and 408 were found to be expressed in at least one tissue, respectively (Table S2, see Section 4.5 for more information on the compilation of the list). Of the expressed splicing-related genes, 38% and 95% were detected to be either 12-h or 24-h rhythmic in at least one of the murine or baboon tissues, respectively. Under the assumption that AS is to some extent a rhythmic process, we would expect a subset of splicing-related genes to be consistently rhythmic across tissues. Thus, we focused on splicing-related genes that were identified as either 12-h or 24-h rhythmic in at least a quarter of the respective tissues (Figure 3A,B). As expected, in both mouse and baboon, most of the splicing-related genes detected to be rhythmic showed 24-h oscillations in transcription, whereas 12-h oscillations were an exception. The five most consistently rhythmic splicing-related genes in murine tissues comprised two members of the hnRNP gene family, *Cirbp* and *Fus*, and three heat shock protein genes, *Hspa1b*, *Hspa5*, and *Hspb1* (Figure 3A). In baboon, the five most consistently rhythmic splicing-related genes were *HNRNPDL*, *ENSPANG00000032278* (a novel orthologous gene to murine genes *Hspa1a* and *Hspa1b*), *INTS1*, *RNF40*, and *XAB2* (Figure 3B). In addition, we identified several splicing-related genes that were consistently rhythmic in both mouse and baboon tissues, including *Hspa1a*/*Hspa1b*/*ENSPANG00000032278*, *Hspb1*, *Rbm45*, and *Srsf5*.

Since the mere co-occurrence of rhythmicity does not necessarily indicate similar oscillatory behaviour, we compared the peak phase and amplitude distributions (Figure 3C,D) and the expression profiles (Figure S3A,B) of our set of candidate splicing-related genes. In terms of phase, we distinguish between circadian time (CT) for mice kept under a DD cycle and zeitgeber time (ZT) for baboons kept under an LD cycle. CT/ZT0 marks the beginning of the light phase and CT/ZT12 marks the beginning of the dark phase. Overall, the peak phases of the individual genes were similar across tissues (mean difference from the circular mean phase per gene: 0.97 h for the mouse and 1.33 h for the baboon), with the exception of *HSPA5* which showed antiphasic oscillations in digestive and excretory baboon tissues compared to its expression patterns in baboon brain tissues (Figure S3B). When comparing peak phases of orthologous splicing-related genes between the diurnal baboon and the nocturnal mouse, we found most of them to be phase-shifted by about 12 h, as expected. In contrast, *Rbm45* and *Cirbp* were only shifted by about 3 h. To attain a broader view of the peak phase distribution of the 24-h rhythmic splicing-related genes, we extended our candidate set to include all 83 genes that were detected to be cycling with a circadian period in at least ten baboon tissues. For this larger set of splicing-related genes, we identified a bimodal distribution of peak phases: The first cluster containing about two thirds of the genes peaked during the subjective day (ZT6.1 ± 0.77) and the second cluster containing about one third of the genes peaked during the subjective night (ZT18.6 ± 1.1) (Figure 3E), indicating a possible control mechanism via components of the molecular clock. Since promoter regions of clock-controlled genes are known to be GC-rich [22], we compared the GC content of the promoter sequences (transcription start site (TSS) ± 1,000 bp) of the two clusters of consistently 24-h rhythmic splicing-related genes with a set of the 18 arrhythmic splicing-related genes that were not detected to be 24-h rhythmic in any baboon tissue. We found the promoters of the set of early peaking splicing-related genes to be significantly more GC-rich than those of the arrhythmic splicing-related genes (Wilcoxon *p*-value = 1.058 × 10^−6^) and the late-peaking splicing-related genes (Wilcoxon *p*-value = 0.002101) (Figure 3F). We further tested for an enrichment of four potential clock transcription factor binding sites (D-box, E-box, E’-box, and Rev response element (RRE)) in the promoter regions of the two clusters of genes. We found the D-box motif to be significantly enriched for the set of early peaking splicing-related genes (adj. *p* = 0.0298), indicating that the observed circadian changes of expression might be due to a direct regulation via first-order clock-controlled transcription factors that bind to D-boxes in a phase-specific manner, such as DBP and TEF [22]. In agreement with this, DBP and TEF were among the top consistently circadian genes in baboon, identified to be 24-h rhythmic in 25 and 26 tissues, respectively, pointing to their potential contribution to the regulation of splicing processes by binding to splicing-related genes. No enrichment for clock transcription factor binding sites was found for the smaller set of late-peaking splicing-related genes, raising the possibility that they might instead be regulated via other clock-controlled genes or through secondary circadian mechanisms.

### 2.3. Rhythmic AS Events are Prevalent in Murine Adipose and Muscle Tissues and are Found in Genes Associated with the Circadian Clock

**Figure 4:**
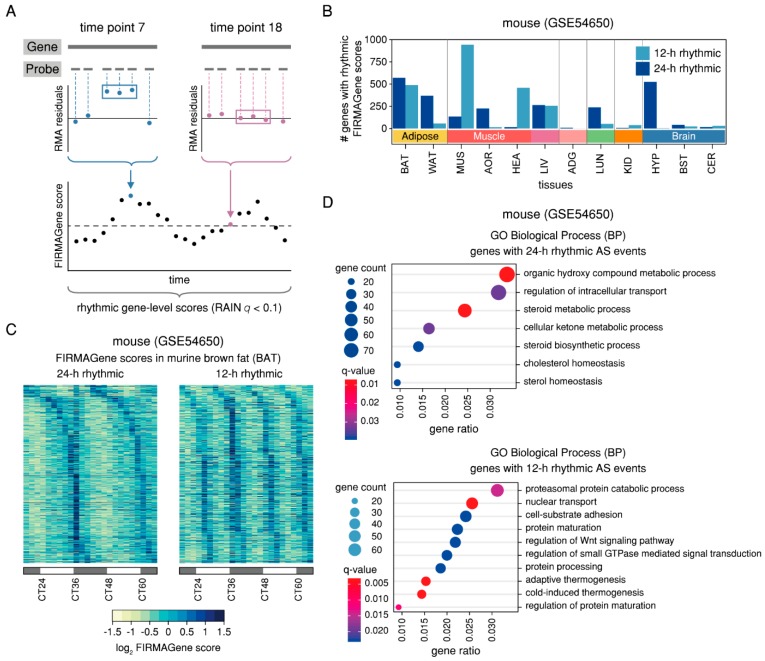
Analysis of whole-transcript microarray data reveals candidate genes with 24-h and 12-h rhythmic AS events in murine tissues. (**A**) Schematic representation of the FIRMAGene analysis to identify candidate genes with rhythmic AS events in the murine microarray dataset. FIRMAGene uses the RMA model to decompose probe-level microarray data into probe effects and expression levels and calculates probe-wise residuals from the RMA fit. Based on the assumption that several adjacent poorly fitting probes for the same exon region that behave differently from the rest (residuals away from zero and in the same direction) can be evidence of potential AS, the algorithm scores the persistence of residuals from the RMA fit, yielding gene-level FIRMAGene scores for each individual time point. (**B**) Number of genes with 24-h rhythmic (dark blue) and 12-h (light blue) candidate AS events in the twelve murine tissues. (**C**) RAIN phase-sorted heatmaps of 24-h rhythmic (left panel) and 12-h rhythmic (right panel) FIRMAGene scores in murine brown fat (BAT). (**D**) Enriched GO terms (Biological Process−BP) for the sets of genes with 24-h rhythmic (upper panel) and 12-h rhythmic (lower panel) candidate AS events in the murine tissues. For genes with 12-h rhythmic AS events, only the first ten GO terms are shown. The complete lists of results can be found in Table S4.

Following the hypothesis that the process of (alternative) splicing may be clock-regulated due to the observed rhythmic expression of splicing-related genes, we would expect to find this rhythmicity mirrored in AS events. While the design of whole-transcript microarray data does not allow for the estimation of transcript-level expression, it can nevertheless be used to identify candidate genes with rhythmic AS events, e.g., by applying the FIRMAGene method that scores potential splicing events based on the persistence of probe-wise residuals from the robust multichip analysis (RMA) fit [23] (Figure 4A). We were interested in identifying genes whose FIRMAGene score varied over time according to a 24-h or 12-h rhythmic pattern, thus indicating a circadian or ultradian regulation of AS. Overall, we identified 2,417 unique genes with 24-h rhythmic and 2,368 unique genes with 12-h rhythmic candidate differential splicing events across all murine tissues (RAIN *q* < 0.1 and relative amplitude ≥ 0.1) (Figure 4B and Table S3). Surprisingly, 12-h rhythmic splicing events not only made up a large proportion of all identified rhythmic events in tissues with many 12-h rhythmic genes like MUS (87%), and BAT (46%), but also in the heart (HEA, 97%) and liver (LIV, 49%) where only few 12-h rhythmic genes had been previously identified (Figure 1B).

One of the candidate genes displaying a 12-h rhythmic FIRMAGene score in BAT was the type 2 diabetes-associated gene *Capn10* which encodes for eight known protein isoforms in humans and has been reported as a target of the splicing factor ZRANB2 [24,25]. Interestingly, *Zranb2* showed a 12-h rhythmic expression pattern in BAT, indicating that its transcriptional oscillations might indeed be propagated to the level of post-transcriptional regulation of one of its target genes (Figure S4D). In adipose tissues, as well as in LIV and lung (LUN), the 24-h rhythmic FIRMAGene scores peaked predominantly at the transition from the light to the dark phase, while in muscle tissues, they peaked in the middle of the subjective day, and in the hypothalamus, in the middle of the subjective night (Figure 4C and Figure S4C). For several genes, rhythmic FIRMAGene scores were identified in more than one tissue, including splicing-related genes such as *Hnrnpa1*, splicing factor gene *Mbnl2*, the core-clock genes *Arntl2* and *Nr1d1*, and the clock-controlled gene *Ciart* (Figure S4A). Notably, the set of genes with 12-h rhythmic FIRMAGene scores in more than one tissue also included several components of the core-clock and clock-controlled genes, such as *Arntl*, *Nr1d2*, *Npas2*, and *Dbp* (Figure S4B). A Gene Ontology (GO) enrichment analysis of the set of candidate genes with 24-h rhythmic FIRMAGene scores revealed several metabolic processes to be among the significantly enriched biological processes (*q* < 0.05), including steroid metabolic processes and sterol homeostasis, as well as the regulation of intracellular transport (Figure 4D and Table S4). Interestingly, candidate genes with 12-h rhythmic FIRMAGene scores were found to be enriched for, i. a., cold-induced and adaptive thermogenesis (Figure 4D), possibly indicating an ultradian control of thermoregulation via rhythmic AS events.

Overall, these findings suggest that oscillating putative AS events with 24-h and 12-h periods are widespread across murine tissues, supporting the hypothesis of a rhythmic regulation of splicing.

### 2.4. Differential 24-h Rhythmic Isoform Pairs in Baboon are Phase-Shifted Across the Daily Cycle

**Figure 5:**
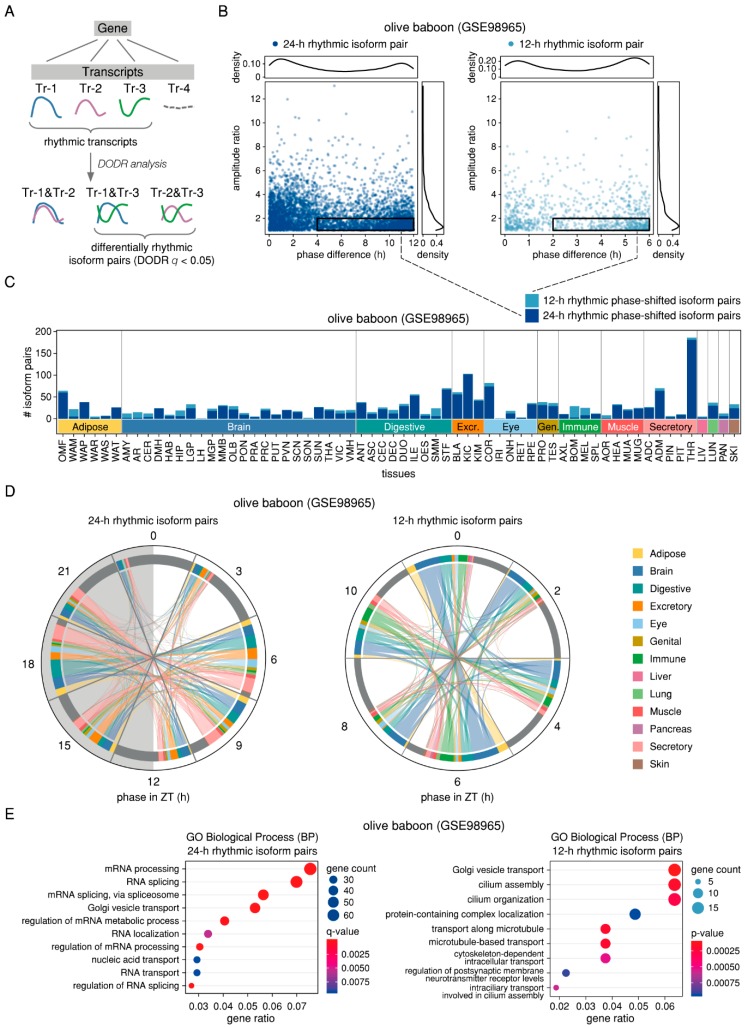
Prevalence of phase-shifted differentially 24-h and 12-h rhythmic splice isoforms in olive baboon tissues. (**A**) Schematic representation of the analysis of differentially rhythmic isoform pairs in RNA-seq data. The rhythmic patterns of all expressed and rhythmic (with the same period) transcripts of the same gene in the same tissue are compared in a pairwise manner using the robust DODR method in order to identify differentially rhythmic isoform pairs with a phase shift in peak expression. (**B**) Amplitude ratio and phase difference of isoform pairs found to be differentially rhythmic (DODR *q* < 0.05) with a period of 24 h (left panel) and 12 h (right panel). Phase-shifted isoform pairs (amplitude ratio < 2 and phase-shift ≥ period/6) are marked by a black rectangle. (**C**) Number of 24-h (dark blue) and 12-h rhythmic (light blue) phase-shifted isoform pairs in the 64 baboon tissues. (**D**) Chord diagrams representing the relations between the two peak phases of expression of 24-h rhythmic (left panel) and 12-h rhythmic phase-shifted isoform pairs (right panel). Peak-phases are clustered in bins of 3 h (left panel) and 2 h (right panel) and coloured according to the type of baboon tissue in which they were detected. (**E**) Enriched GO terms (Biological Process—BP) for the sets of genes that exhibited 24-h rhythmic (left panel) and 12-h rhythmic phase-shifted isoform pairs in the baboon tissues. For visualisation, GO terms were manually curated to remove redundant categories. The complete lists of results can be found in Table S4.

Given the differences found between rhythmic features at gene- and at transcript-level in baboon (Figure 2), the consistently 24-h rhythmic expression of splicing-related genes in both mouse and baboon (Figure 3), and the observed rhythmicity of putative AS events in mouse (Figure 4), we used the transcript-level baboon RNA-seq data to directly search for isoforms of the same gene that show phase-shifted rhythmic expression patterns. To detect cases of differential rhythmicity of transcripts, we applied the DODR method which tests for amplitude and phase changes between two conditions of one rhythmic feature [26]. In particular, we compared each rhythmic transcript (RAIN *p* < 0.005 and relative amplitude ≥ 0.1) to all other rhythmic transcripts (RAIN *p* < 0.05 and relative amplitude ≥ 0.1) of the same gene with the same period in the same tissue in a pairwise manner (Figure 5A). Taking all tissues together, we obtained 16,120 pairs of 24-h rhythmic and 2576 pairs of 12-h rhythmic transcripts of which 4546 pairs (2,096 genes) and 888 pairs (741 genes) were differentially rhythmic (DODR *q* < 0.05), respectively. On average, a candidate gene with differentially rhythmic transcripts was detected in 1.9 tissues for 24-h rhythmic isoform pairs and in 1.1 tissues for 12-h rhythmic isoform pairs (Figure S5A). Differential rhythmicity can be due to changes in either amplitude or phase of the rhythmic features, or both. We were particularly interested in identifying rhythmic transcripts pairs with pure phase shifts in the same tissue, hypothesising that they might represent functionally distinct splice isoforms of the same gene that are expressed at different times of the day. Hence, we filtered for isoform pairs with an amplitude ratio smaller than 2 that were phase-shifted by at least a sixth of their period length, resulting in a total of 1495 (1201 unique isoform pairs, 969 unique genes) and 320 (313 unique isoform pairs, 289 unique genes) phase-shifted isoform pairs with 24-h and 12-h rhythmic expression patterns, respectively (Figure 5B,C and Table S5).

An analysis of the phase distribution and the phase difference of all phase-shifted 24-h rhythmic isoform pairs revealed two clusters of transcripts that exhibited a phase-shift greater than 10 h and that showed two peaks of expression between ZT 4–10 and between ZT 15–21 (Figure S5B). We further compared the relations between the two peak times of a phase-shifted isoform pair and found that the majority of 24-h rhythmic phase-shifted transcripts were indeed expressed at opposing times of the daily cycle, with one transcript peaking in the middle of the subjective day and the other in the middle of the subjective night (Figure 5D). For the set of 12-h rhythmic phase-shifted isoform pairs, the peak times were distributed over the 12-h ultradian cycle, with most transcripts exhibiting phase-shifts greater than 4 h (Figure S5B and Figure 5D).

A GO enrichment analysis of the whole set of candidate genes with 24-h rhythmic phase-shifted isoforms revealed RNA processing, RNA splicing, Golgi vesicle transport, the regulation of mRNA metabolic processes, and RNA localisation to be among the significantly enriched biological processes (*q* < 0.05) (Figure 5E and Figure S5C and Table S4). Interestingly, Golgi vesicle transport was also found to be enriched for the set of candidate genes with 12-h rhythmic phase-shifted isoforms (*p* < 0.05) (Figure 5E). The prevalence of RNA splicing among the enriched processes hints at a reciprocal regulation between the observed circadian rhythmicity of genes encoding for spliceosome components and splicing factors and the tissue-spanning production of phase-shifted 24-h rhythmic isoforms of genes involved in the splicing process. Several candidate genes displayed 24-h rhythmic phase-shifted isoform pairs in multiple tissues, including the splicing factor gene *PCBP2* (eight tissues), the RNA binding protein gene *NELFE* (seven tissues), *CIRBP* (six tissues), and the scaffold protein gene *RACK1* (five tissues) (Figure S5C,D). Interestingly, both PCBP2 and NELFE have been identified as oncogenes [27,28]. CIRBP is known as a mediator of cancer-related inflammation and has been reported to act both as a tumour suppressor and a tumour promoter, depending on the cell type and cancer stage [29], while RACK1 plays an important role in the regulation of key signalling pathways in cancer [30]. In addition to the gene-level GO enrichment analysis, we searched for experimentally validated functionally distinct splice isoforms (FDSIs) in healthy tissues [31] among our list of candidate differentially rhythmic splice isoforms. Of the 23 human genes with FDSIs meeting the criteria of Bhuiyan and colleagues, we found ten orthologous olive baboon genes with differentially 24-h rhythmic isoform pairs in at least one tissue (*CFLAR*, *EIF4G1* and *EIF4G2*, *KLF6*, *MADD*, *MST1R*, *PML*, *PRMT5*, *STIM2*, and *SUN1*). Of these, the eukaryotic initiation factor gene *EIF4G2*, the splicing regulator gene *PRMT5*, and the tumour suppressor genes *KLF6* and *PML* had purely phase-shifted isoform pairs.

Thus, our findings regarding phase-shifted differentially rhythmic splice isoforms identified in a variety of baboon tissues suggest a time-of-day dependent regulation of mRNA splicing through alternative transcript usage of, i. a., splicing-related genes.

### 2.5. Genes with Phase-Shifted Splice Isoforms in a Human Cell Line Model of Colorectal Cancer Progression are Enriched for mRNA Splicing and Have a Putative Impact on Cell Survival and Migration

**Figure 6:**
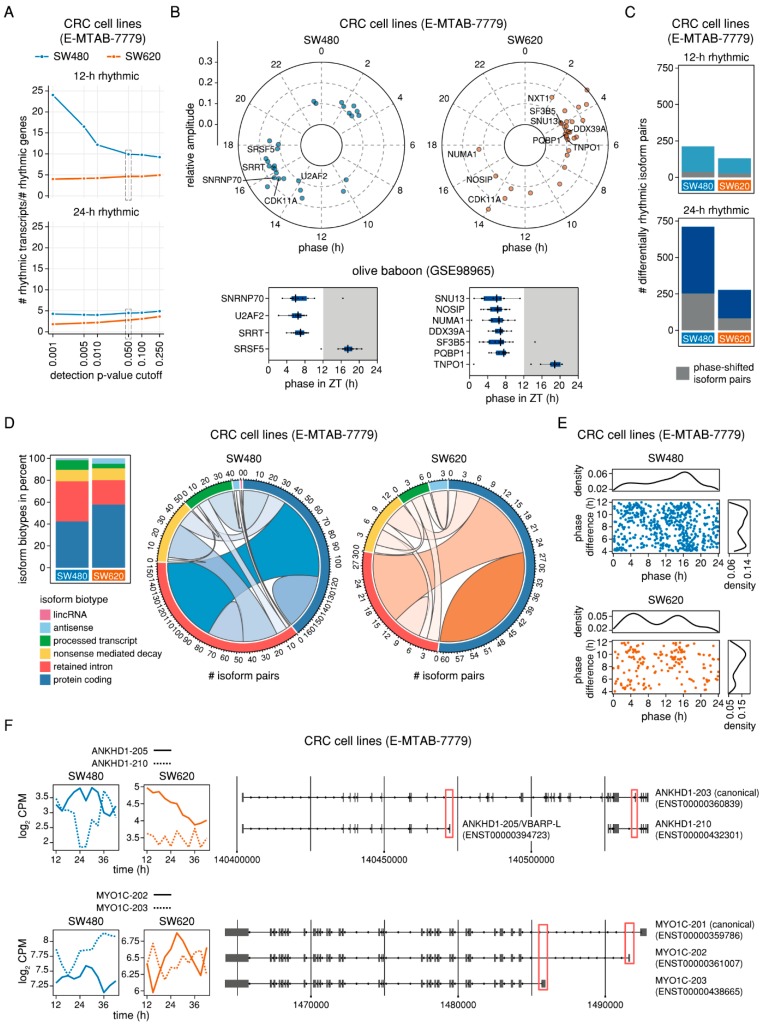
Phase-shifted splice isoforms in human CRC cell lines. (**A**) Ratio of 12-h rhythmic (upper panel) and 24-h rhythmic (lower panel) transcripts and genes for SW480 (blue) and SW620 (orange) for different RAIN *p*-value cut-off levels. (**B**) Distribution of peak phases of 24-h rhythmic splicing-related genes in the CRC cell lines SW480 (left upper panel) and SW620 (right upper panel) and their respective orthologous genes in the baboon tissues (lower panels). Splicing-related genes that were either shared between both cell lines or for which 24-h rhythmic orthologous baboon genes were identified, are labelled by name. The grey area represents the subjective night from ZT12 to ZT24 (**C**) Number of 12-h (upper panel) and 24-h rhythmic (lower panel) differentially rhythmic isoform pairs in the CRC cell lines. The proportion of phase-shifted isoform pairs is marked in grey. (**D**) Transcript biotypes of 24-h phase-shifted isoform pairs in percent (left panel) and chord diagrams representing the types of isoform pairs for SW480 (middle panel) and SW620 (right panel). (**E**) Phase distribution and pairwise phase-difference of phase-shifted differentially 24-h rhythmic isoform pairs in SW480 cells (upper panel, blue) and SW620 cells (lower panel, orange). (**F**) Expression (log_2_ CPM) of the transcripts ANKHD1-205 and -210 (upper left panel) and MYO1C-202 and -203 (lower left panel) in SW480 (blue) and SW620 (orange) and visualisation of the genomic structure of *ANKHD1* (upper right panel) and *MYO1C* transcripts (lower right panel). Differences in the exonic composition are marked with red rectangles.

Deregulations in splicing are often implicated in human disease, including cancer where frequent alterations in splicing events and mutations in splicing factor genes have been reported [4,32,33,34]. Interestingly, several of our candidate genes with phase-shifted isoform pairs in multiple baboon tissues were oncogenes and tumour suppressors. Thus, to investigate whether the rhythmic regulation of isoform expression observed in normal mammalian tissues would be affected in tumorigenesis, we profiled the circadian transcriptome of two human CRC cell lines by RNA-seq and estimated gene- and transcript-level expression values as previously described (Figure 1A). We chose cell lines derived from a primary carcinoma (SW480) and a lymph node metastasis (SW620) of the same patient known to have different clock phenotypes [20] as a model system. Overall, we found 1343 24-h rhythmic genes and 6,003 24-h rhythmic transcripts in SW480 cells and 1934 24-h rhythmic genes and 5,395 24-h rhythmic transcripts in SW620 cells (RAIN *p* < 0.05 and relative amplitude ≥ 0.1) (Figure S6A). As previously observed in mouse and baboon tissues, 12-h rhythms in transcription were less prevalent (Figure S6A). Interestingly, the ratio of rhythmic transcripts to rhythmic genes was higher for SW480 for both tested periods, indicating that despite the higher number of genes detected as rhythmic in SW620, the number of individual oscillating isoforms was higher in the primary tumour cell line (Figure 6A). In both SW480 and SW620 cells, splicing-related genes displayed 24-h rhythms in expression that were specific for the respective cell line except for the cycling dependent kinase gene *CDK11A* that was identified as oscillating for both (Figure S6B). In SW480, a bimodal distribution of peak phases was observed in line with our findings in baboon tissues (Figure 6B). However, when comparing the phases of individual splicing-related genes to those of their orthologous baboon genes (*SRSF5*, *SNRNP79*, *SRRT*, and *U2AF* in SW480 and *DDX39A*, *NOSIP*, *NUMA1*, *PQBP1*, *SF3B3*, *SNU13*, and *TNPO1* in SW620), the phases of the human genes did not cluster in the same way as the baboon genes did (Figure 6B). This could be due to differences between tissues or result from alterations in clock-controlled transcription associated with tumour progression.

We further detected 253 and 83 phase-shifted 24-h rhythmic pairs and 36 and 26 phase-shifted differentially 12-h rhythmic pairs for SW480 and SW620 cells, respectively (Figure 6C and Figure S6C and Table S6). Given the small size of the 12-h rhythmic sets, we only conducted the subsequent analyses for the set of 24-h rhythmic phase shifted isoforms. In SW480 cells, 30% of the phase shifted isoform pairs consisted of a protein coding transcript and a transcript with a retained intron, followed by pairs of two protein-coding transcripts (19.8%), pairs of two transcripts with retained introns (12.6%), and pairs of a transcript with a retained intron and a nonsense-mediated decay (NMD) transcript (12.3%) (Figure 6D). In SW620 cells, the largest set were pairs of two protein-coding transcripts (42%), followed by pairs consisting of a protein-coding transcript and a transcript with a retained intron (21.7%) (Figure 6D).

In SW480 cells, we detected a peak of expression for transcripts with phase-shifted isoform pairs at ~16.5 h, whereas the distribution for SW620 cells was bimodal, with peaks at ~5 h and ~15 h (Figure 6E). A GO analysis revealed that the genes with phase-shifted isoform pairs were enriched for, i. a., lipoprotein-related processes, as well as for cytokinesis and the negative regulation of RNA splicing in SW480 cell lines, and for (alternative) RNA splicing, post-transcriptional regulation of gene-expression, and the regulation of translation in SW620 cells (Figure S6E and Table S4). The prevalence of splicing-related processes for both cell lines mirrors the results of the enrichment analysis for the baboon tissues and is in line with results from previous studies that have reported extensive AS of splicing-related genes in cancer [35,36]. 16 genes from the list of splicing-related genes (Table S2) were found among the candidates in SW480 cells (*CLK4*, *DDX3X*, *GRSF1*, *HNRNPK*, *HNRNPL*, *INTS6L*, *MOV10*, *NONO*, *NXF1, PCBP2*, *RBM3*, *RBM39*, *SRRT*, *TAF15*, *U2AF*, *ZNF131*) and eight genes were found in SW620 cells (*CCAR2*, *CELF1*, *CIRBP*, *DDX17*, *HNRNPC*, *PABPC1*, *RBM4*, *SRSF2*) (Figure S6D). Only three of the splicing-related candidate genes in SW480 (*RBM39*, *SRRT*, and *U2AF2*) and none in SW620 had previously been found to be oscillating at gene-level (Figure S6B), indicating that their transcript-level rhythmic expression was masked due to the phase-shifted expression of their individual transcripts. Except for *CCAR2*, *CELF1*, and *RBM4*, not all transcripts pairs of splicing-related genes were annotated to be protein-coding which makes it unclear whether the observed rhythmic AS has a functional effect on cellular physiology or whether it is unproductive.

In light of our previous findings and the known importance of AS events in carcinogenesis and tumour-progression, we checked for cancer-related splice isoforms among our candidate genes for which both transcripts were annotated as protein-coding, such as *ANKHD1* in SW480 and *MYO1C* in SW620 (Figure 6F). ANKHD1 is an ankyrin repeat and KH domain-containing protein for which two functional AS variants have been characterised: In contrast to the canonical isoform, VBARP-L (11 exons) and VBARP-S (9 exons) both contain only a single ankyrin repeat motif and lack the signature KH domain. [37]. The 24-h rhythmic transcript ANKHD1-205 in SW480 encodes for VBARP-L, whereas the ~10.5 h phase-shifted transcript ANKHD1-210 consists of eight exons that encode for the KH domain and do not overlap with the exons of ANKHD1-205 (Figure 6F). *MYO1C* is a member of the unconventional myosin gene family and produces three AS isoforms through N-terminal splicing which differ in their functions and their nucleo-cytoplasmic partitioning [38]. The 24-h rhythmic transcript MYO1C-202 in SW620 encodes for the isoform MYO1C^C^ which localises predominantly to the plasma-membrane and is involved in cell migration and signal transduction, whereas the ~7.4 h phase-shifted transcript MYO1C-203 encodes for the isoform MYO1C^B^, also known as nuclear myosin I (NMI) or MYO1C^16^ which localises mostly to the nucleus where it is involved in transcription, mRNA maturation, and chromatin remodelling [38] (Figure 6F). Notably, the expression patterns of *ANKHD1* and *MYO1C* transcripts differ between the cell lines (Figure 6F), indicating that they are indeed specific for different stages of tumour progression. Altogether, our findings concerning oncogenes and tumour suppressor genes among the candidates with putative rhythmic AS events in healthy baboon tissues are reinforced by the results of the analysis in the human CRC progression model, uncovering potential cancer-relevant rhythmically expressed isoforms.

## 3. Discussion

AS is an essential pre-mRNA processing mechanism that enables the functional diversification of the proteome via the production of several distinct protein isoforms from a single gene. In this study, we aimed to elucidate whether the process of AS might be temporally regulated over the course of the day at the organismal level, potentially leading to rhythmic expression patterns of individual splice isoforms. For this purpose, we developed a workflow for the genome-wide identification of AS events in circadian whole-transcript microarray data and the detection of differentially rhythmic isoforms of the same gene in circadian RNA-seq data (Figure 1A). Using the most comprehensive mammalian circadian dataset of a diurnal primate currently available, we compared the number and the rhythmic properties of 24-h and 12-h rhythmic features identified either at gene- or at transcript-level in 64 tissues from the olive baboon. In addition to the prevalence of 24-h transcriptional rhythmicity in mammalian tissues, the existence of higher-order harmonics of circadian gene expression has been shown for mouse liver in vivo [17], and recently also for murine fibroblast cells in vitro [19]. Hence, we also tested for ultradian rhythms with a 12-h period. We found that while the correlation between the number of rhythmic genes and the number of rhythmic transcripts was roughly linear, the identity of rhythmic genes differed depending on whether the input expression data was based on individual transcript counts or on summarised gene counts. We further observed that some genes were classified as rhythmic even though their individual transcripts were arrhythmic. Conversely, some genes were not defined as rhythmic, despite having oscillatory transcripts that displayed phase-shifted patterns. Interestingly, the sets of rhythmic transcripts displayed higher amplitudes than the sets of rhythmic genes; moreover, features identified as rhythmic on both transcript- and gene-level had lower *p*-values in the rhythmicity analysis than features identified only on gene-level. Thus, our results demonstrate that a transcript-level rhythmicity analysis might on the one hand potentially contribute to the improvement of rhythm detection on gene-level and on the other hand yield novel results concerning oscillating splice isoforms which would otherwise be overlooked.

Splicing-related genes have previously been found to oscillate with circadian expression patterns in murine liver [9] and human colorectal cancer cell lines [10]. In the present study, we identified a set of more than 80 consistently 24-h rhythmic splicing-related genes in baboon tissues that displayed a bimodal distribution of peak phases with two thirds of the genes peaking during the subjective daytime (early genes) and one third of the genes peaking during subjective nighttime (late genes). Our findings are in line with the results of McGlincy and colleagues who also identified two clusters of peak phases during the subjective day and night [9]. We further found evidence for a possible clock regulation of the set of early-peaking splicing-related genes whose promoter regions were significantly more GC-rich than those of late-peaking and arrhythmic splicing-related genes. They were also enriched for the D-box motif, a potential clock transcription factor binding site [39]. GC-rich motifs have been found to be overrepresented in promoter regions of clock-controlled genes [22]; hence, our results point to a possible regulation of the early-peaking splicing-related genes via first-order D-box binding clock-controlled transcription factors such as *DBP* and *TEF* [39]. According to the TRANSFAC database of eukaryotic transcription factors and their genomic binding sites, both the early-peaking gene *DHX30* and the late-peaking gene *SYNCRIP* are targets of DBP [40,41]. Several of the consistently 24-h rhythmic splicing-related genes were also found to oscillate in murine tissues with approximately 12-h phase shifts when compared to the baboon: the hnRNP coding gene *Fus* and the splicing factor gene *Srsf5*, both of which peak during the active phases of the animals, and the heat shock protein gene *Hspb1* that peaks in the rest phase. Fus is a neurodegeneration-associated protein that binds to distinct RNA sites of its target genes to regulate the inclusion of alternative exons and that has recently been found to be transcriptionally regulated by core-clock component Nr1d1 and to modulate the expression of core-clock genes *Per2* and *Cry1* [42,43]. In contrast, both *Hspb1* and *Srsf5* have been reported to be regulated via temperature variations: While Hspb1 expression has been associated with the efficiency of splicing recovery after heat shock [44], transcript and protein levels of the splicing factor Srsf5 have been shown to be cold-inducible in mammalian cells cultured at 32 °C [45]. Interestingly, the cold-inducible splicing factor SRSF5 also displayed 24-h rhythmic patterns in transcription in human SW480 cells kept under constant conditions, indicating that its oscillations are not solely due to changes in the internal body temperature of animals. A notable exception to the expected ~12-h shift between oscillations in a diurnal and a nocturnal animal was the cold-inducible hnRNP encoding gene *Cirbp*: In mouse, *Cirbp* peaks in the middle of the subjective day, coinciding with a trough in the core body temperature of male C57Bl/6 mice [46], supporting the hypothesis of a temperature-dependent control of *Cirbp* [47,48]. Yet, the morning peak of expression of *CIRBP* at ~ZT3 in baboon overlapped with the time at which the body temperature of the animals reached its peak (~37.8 °C) [16], indicating that *Cirbp* control mechanisms might differ between diurnal and nocturnal mammals. It has to be noted that in vivo, 24-h rhythms in transcription may be due to direct circadian regulation via components of the molecular circadian clock or clock-controlled transcription factors, or due to secondary circadian effects, such as diurnal changes in body temperature [49] or cortisol levels [50]. For instance, AS of the splicing factor gene *U2af26* has been shown to be temporally controlled via temperature changes [51] and the cortisol-inducible splicing factor SRSF3 promotes the splicing of human glucocorticoid isoform GRα which transcriptionally regulates *RACK1* by binding to its promoter [52].

Under the assumption that the 24-h rhythmic expression patterns of *trans*-acting splicing-related genes might be propagated to the transcriptional output of their target genes, we looked for evidence of rhythmic splicing events on a genome-wide level for two mammalian model organisms, the nocturnal mouse and the diurnal baboon. Taking into account the technical design of the circadian whole-transcript array data from mouse, we found candidate genes with putative rhythmic AS events with either 24-h or 12-h periods to be prevalent in adipose and muscle murine tissues. Interestingly, many of the candidate genes with rhythmic AS events identified in multiple tissues were associated with the circadian clock. While the study by Zhang and colleagues [15] also contains 48-h time-series RNA-seq data for the same murine organs, the sampling resolution of 6 h (instead of 2 h for the microarrays) does not suffice to identify 12-h or 24-h rhythmic features with confidence and thus could not be used for the purpose of our study.

Remarkably, our transcript-level analysis of the baboon RNA-seq data revealed a wide array of differentially 24-h rhythmic transcripts of the same gene whose oscillatory expression patterns displayed similar amplitudes but peaked at opposing times of the daily cycle, indicating a rhythmic control of AS events. This genome-wide distribution of daily and nightly isoform peaks suggests a possible distinct functional role of the involved transcripts that might depend on their expression level at a specific time-of-day. At gene-level, phase-shifted isoform pairs were found to be significantly enriched for RNA processing and mRNA splicing processes. This suggests a reciprocal interplay between the observed circadian expression patterns of splicing-related genes on the one hand and the time-of-day-dependent production of splice isoforms on the other hand. In addition, Golgi vesicle transport was found to be enriched for both the set of candidate genes with 24-h and with 12-h rhythmic phase-shifted isoforms, suggesting a complex temporal regulation of the secretory pathway that may possibly be regulated via circadian and ultradian splice isoform production. AS events have recently been shown to affect the secretory pathway [53] and a circadian control of procollagen transport from ER-to-Golgi and Golgi-to-plasma membrane has been reported in mice [54]. Among the genes with 24-h rhythmic phase-shifted isoform pairs in multiple baboon tissues, we identified several oncogenes and cancer-related genes, including *PCBP2*, *NELFE*, *CIRBP*, and *RACK1*. We further compared our candidate rhythmically spliced baboon genes to a list of genes with known functionally distinct isoforms that have been experimentally validated in human. Of these, the genes *EIF4G2*, *PRMT5*, and the tumour suppressor genes *PML* and *KLF6* expressed phase-shifted isoform pairs in baboon tissues. Interestingly, AS of human *KLF6* results in the dominant negative splice isoform KLF6-SV1, which has been identified as a key driver of breast cancer and prostate cancer metastasis by promoting cell survival, migration, and invasion [55,56]. However, it still remains to be elucidated whether the specific isoforms and their function are conserved between human and baboon.

In view of the known significance of alternatively spliced isoforms in cancer and tumour progression [32,33] which is further reinforced by our discovery of oncogenes and tumour suppressors among the candidate rhythmically spliced genes, we extended our study by including a novel RNA-seq dataset of human CRC cell lines SW480 (primary tumour) and SW620 (metastasis) that originate from the same patient. Both SW480 and SW620 cells displayed 24-h rhythms in transcription of splicing-related genes as well as phase-shifted isoform pairs that were largely specific for the respective cell line. As previously observed in baboon, the genes with 24-h rhythmic phase-shifted isoform pairs were enriched for splicing processes and included various splicing-related genes, e.g., *NONO* and *PCBP2* in SW480 and *CELF1* and *CIRBP* in SW620 cells. *NONO*, *PCBP2*, and *CIRBP* also displayed phase-shifted isoforms in multiple baboon tissues, indicating that rhythmic AS of these genes might not only be conserved across tissues but also across different mammalian species. The transcription factor NONO is known to bind to the core-clock protein PER and to antagonise its activity [57] and has furthermore been reported to act as a coupling element between the circadian clock and the cell cycle [58]. CELF1 has been reported to promote the expression of pro-tumourigenic genes and to be a critical regulator of cancer biological processes, such as proliferation, cell survival, and epithelial-mesenchymal transition (EMT) [59,60]. In line with these findings, several of the phase-shifted protein-coding transcripts in the CRC cell lines, e.g., *ANKHD1* and *MYO1C* transcripts, were found to be associated with cancer-related biological processes such as cell survival and migration, hinting at a possible role of rhythmic AS in CRC tumour progression. ANKHD1 is overexpressed in acute leukaemia [61] and its VBARP isoforms have been reported to have an antiapoptotic effect and to play a role in cell survival pathways [37]. MYO1C^A^, an isoform of MYO1C, is involved in prostate cancer cell migration [62] and it has been speculated that alterations of MYO1C at the plasma membrane might affect the metastatic potential of tumour cells [63]. Thus, it is conceivable that a temporal regulation of functionally distinct isoforms might enable CRC cells to produce protein isoforms at specific times of the day that facilitate their malignant transformation.

In summary, circadian splicing-related genes and rhythmic AS events with a 24-h period (and to a lesser extent a 12-h period) appear to be widespread across mammalian tissues, supporting the hypothesis of a circadian regulation of the splicing process. Several of the candidate differentially spliced genes encode for functionally distinct protein isoforms that might influence cellular processes in a time-of-day-dependent manner. Nonetheless, further experimental datasets with a higher coverage and/or longer reads and containing a sufficient number of replicates per condition (which are usually not part of the design of high-throughput circadian transcriptome studies [64]) will be necessary for the subsequent exploration of our results. We partially mitigated this problem by focusing on candidate genes for which phase-shifted isoform pairs could be found in more than one tissue, thus strengthening our confidence in the putative circadian AS event. However, AS is known to be tissue-specific [3], a fact that is likely also true for circadian AS [9]. Thus, the experimental design of future studies on rare tissue-specific rhythmic AS events should implement further measures for the accurate detection of alternative splice isoforms, either by including additional time point replicates or by increasing coverage and/or RNA-seq read length. Moreover, information about the functional impact of specific isoforms is still sparse to date [65], especially for less-extensively studied model organisms such as the olive baboon, thus preventing a functional annotation at transcript-level. Future experimental studies that aim at targeted isoform depletion of a phase-shifted transcript pair might yield more information concerning the functional effect of rhythmic AS and ascertain whether the affected splice isoforms are indeed functionally relevant and thus contribute to the postulated diversity of the proteome. Nonetheless, our systems-level analysis across different tissues and organisms provides compelling evidence for rhythmic AS and its potential role in the temporal diversification of the mammalian proteome. Thus, it is conceivable that diverse splice isoforms expressed at different times of the day will lead to a time-dependent function of proteins and that failures in this temporal control may impact on the normal regulation of cellular processes and ultimately drive malignancy.

## 4. Materials and Methods

### 4.1. Cell Culture

Human colorectal carcinoma cell lines SW480 and SW620 were purchased from the ATCC. Cells were maintained in DMEM low glucose (Lonza, Walkersville, MD, USA) culture medium supplemented with 10% FBS (Life technologies, Inc., Wilmington, DE, USA), 1% penicillin-streptomycin (Life technologies, Inc., Wilmington, DE, USA), 1% HEPES (Life technologies, Inc., Wilmington, DE, USA) and 2 mM Ultraglutamine (Lonza, Walkersville, MD, USA) at 37 °C in a humidified atmosphere with 5% CO_2_.

### 4.2. Sample Preparation

SW480 and SW620 cells were seeded in triplicates in 6-well plates with a density of 1–2 × 10^6^ cells one day prior to the experiment. On the next day, cells were synchronised by changing the medium. Sampling was started 12 h after synchronisation and samples were taken every 3 h for a time-series of 30 h and prepared for RNA extraction. Total RNA was isolated using the RNeasy Mini Kit (Qiagen, Valencia, CA, USA) including 15 min DNase I digestion according to the manufacturer’s instructions. Prior to the purification procedure, medium was discarded and cells were washed twice with PBS and lyzed in RLT buffer (Qiagen, Valencia, CA, USA). RNA was eluted in 30–50 µL RNase-free water. The final RNA concentration was determined with a NanoDrop 1000 spectrophotometer (Thermo Fisher Scientific, Wilmington, DE, USA). RNA was stored at −80 °C until use. mRNA libraries were generated from high quality RNA (average RNA quality number (RQN) of 10.0) and prepared using the TruSeq Stranded mRNA Sample Preparation Kit (Illumina, San Diego, CA, USA) according to the low sample protocol guidelines and sequenced on an Illumina NextSeq 500 platform to an average depth of 74 M (min. 49 M, max. 129 M) 75 bp paired-end reads at the European Molecular Biology Laboratory (EMBL) GeneCore Facility (EMBL, Heidelberg, DE).

### 4.3. Processing Microarray and RNA-Seq Transcriptome Data

For the murine microarray dataset (GSE54650), raw expression data was downloaded from the Gene Expression Omnibus (GEO) database and preprocessed using the Robust Multichip Average (RMA) [66,67] function from the oligo R package version 1.42.0 [68]. Transcript clusters were annotated using Affymetrix mogene10 annotation data (https://bioconductor.org/packages/release/data/annotation/html/mogene10sttranscriptcluster.db.html, version 8.7.0). For the human CRC cell lines RNA-seq dataset, quality control was performed using FastQC version 0.11.7 [69] and residual adapter sequences were trimmed from the reads using Trimmotatic version 0.38 [70] with TruSeq3-PE-2.fa adapter sequences (default parameters). Only paired-end reads were retained for the downstream analyses. Raw data have been deposited in the EBI’s ArrayExpress database under the accession number E-MTAB-7779.

Single-end sequencing reads for the olive baboon dataset (GSE98965) were downloaded from the European Nucleotide Archive (ENA). Sequencing reads were aligned to the human genome (Homo_sapiens.GRCh38, Ensembl release 92), and the *Papio anubis* genome (Panu_3.0, Ensembl release 93) respectively, using STAR aligner version 2.6.0a [71] with default parameters and the option —quantMode TranscriptomeSAM. The TranscriptomeSAM option generates both a genome and a transcriptome alignment by first aligning reads to the genome and then searching for concordance between the genomic alignment and annotated transcripts. If a genomic alignment is mapped to several transcriptomic coordinates, it is projected to all of them. The sample of the olive baboon amygdala (AMY) taken at time point ZT 8 had a very low mapping rate (<1%) and was therefore excluded from further analyses. Based on the STAR transcriptome alignment, transcript-level abundance was quantified in units of transcripts per million (TPM) using Salmon version 0.10.2 [72] in alignment-based mode with default parameters and the —seqBias option. Salmon considers all of the projected alignment positions of a read and allocates it probabilistically by maximising the joint likelihood of all the observed data. The tximport R package version 1.6.0 [73] was used to import and scale the resulting count tables by first multiplying TPM by feature length and then scaling up to the library size (lengthScaledTPM) resulting in both transcript-level (txOut = TRUE, based on Ensembl Gene IDs) and summarised gene-level (txOut = FALSE, based on Ensembl Transcript IDs) count estimates. For each sample, normalisation factors to scale the raw library sizes were calculated using the trimmed mean of M-values (TMM) method and counts were log2-transformed using the *cpm* function from the edgeR R package version 3.20.9 [74]. For the baboon tissues, lowly expressed genes and transcripts were filtered out, retaining only features with at least 0.5 counts per million (CPM) on average over all time points per tissue. For the human CRC cell lines, all features with at least 0.5 CPM on average over all time points in at least one of the two cell lines were retained. Counts were then renormalised using only the selected features.

### 4.4. Rhythmicity Analysis

Rhythmicity analysis was performed on the absolute RMA-preprocessed transcript expression values for the array data and on the transcript- and gene-level CPM values for the sequencing data. 12-h and 24-h rhythmicity was assessed using the RAIN R package version 1.12.0 [75]. Phase and relative amplitude were determined by fitting a robust harmonic regression using the HarmonicRegression R package version 1.91 [76] with a period of 12 h and 24 h, respectively. Circular mean and median of peak phases were calculated with the R package circular (0.4-93) [77].

In the case of multiple transcript clusters annotated with the same Ensembl Gene ID in the array data, only the transcript cluster with the lowest RAIN *p*-value per tissue was retained. For both the array and the sequencing data, genes and transcripts with relative amplitude < 0.1 were excluded from the set. The *p*-values of the remaining genes and transcripts of the sequencing data were FDR-corrected using the Benjamini-Hochberg procedure. Significance for 12-h and 24-h rhythmic features was bounded by *q* < 0.05 for the mouse array data sampled at 2-h intervals for 48 h, by *p* < 0.005 for the baboon sequencing data sampled at 2-h intervals for 24 h and by *p* < 0.05 for the CRC cell line sequencing data sampled at 3-h intervals for 30 h. Tissue-wise area-proportional Venn diagrams of the logical relations between 12-h and 24-h rhythmic gene and transcript sets of the olive baboon were visualised using the R package eulerr version 5.0.0 [78].

### 4.5. Compilation of a List of Splicing-Related Genes

A list of 426 human spliceosome and splicing-related genes was compiled by aggregating elements from the following sources: 1) a list of 254 spliceosome components and splicing regulators from a previous publication of our group [10], and 2) 404 splicing factor genes from a study by Seiler and colleagues Seiler, Peng, Agrawal, Palacino, Teng, Zhu, Smith, Caesar-Johnson, Demchok and Felau [34]. Mapping these genes to their orthologues using Ensembl BioMart (Ensembl Genes 95) [79] resulted in 429 splicing-related genes for baboon and 451 splicing-related genes for mouse (Table S2).

### 4.6. Prediction of Clock Transcription Factor Binding Sites

Promoter sequences were extracted for the two sets of splicing-related genes detected to be 24-h rhythmic in at least ten of the baboon tissues that showed an early and a late peak of expression, respectively, and for a set of arrhythmic splicing-related genes. For each consistently 24-h rhythmic splicing-related gene, all associated 24-h rhythmic transcripts were identified, while for the set of arrhythmic splicing-related genes, the associated transcripts were filtered for those expressed in at least one baboon tissue but not detected to be 24-h rhythmic (*p* > 0.05) in any tissue. Sequences of promoter regions ± 1000 bp of the TSSs from Ensembl BioMart (Ensembl Genes 95) [79] were extracted using bedtools [80]. Duplicate sequences were removed. The enrichment of four potential clock transcription factor binding sites (D-box, E-box, E’-box, and RRE) in the promoter regions of the sets of early and late-peaking splicing-related genes was tested using the Analysis of Motif Enrichment (AME) tool of the MEME Suite version 4.12.0 [81] based on position-specific scoring matrices (PSSMs) [9], employing the average odds scoring method and one-tailed Fisher’s exact test with shuffled input sequences as control.

### 4.7. Detection of Rhythmic AS in Circadian Whole-Transcript Microarray Data

Candidate genes with potential AS events in the murine microarray data were predicted using the FIRMAGene method that was specifically developed for the Affymetrix Gene 1.0 ST platform and that can be applied in the absence of replicates [23]. Using the RMA model, FIRMAGene decomposes probe-level microarray data into probe effects and expression levels and calculates the departure from the model through probe-wise residuals from the RMA fit. Based on the assumption that several adjacent poorly fitting probes for the same exon region that behave differently from the rest can be evidence of potential AS, the algorithm scores the persistence of residuals from the RMA fit. Gene-level FIRMAGene scores were calculated for each time point using the FIRMAGene R package version 0.9.8 in the Aroma platform as implemented in the aroma.affymetrix R package version 3.1.1 [82] and a custom chip definition file (CDF) for MoGene (MoGene-1_0-st-v1,mm9.cdf, http://bioinf.wehi.edu.au/folders/firmagene/). Relative amplitudes of the FIRMAGene scores were determined by fitting a robust harmonic regression using the HarmonicRegression R package version 1.91 [76] with a period of 12 h and 24 h, respectively. Scores with relative amplitude < 0.1 were excluded. The RAIN *p*-values of amplitude-filtered scores were FDR-corrected using the Benjamini-Hochberg procedure and significance for 12-h and 24-h rhythmic FIRMAGene scores was bounded by *q* < 0.1. Tissue-wise intersections between sets of genes with rhythmic FIRMAGene scores were visualised using the UpSetR R package version 1.3.3 [83].

### 4.8. Detection of Differentially Rhythmic Splice Isoforms in Circadian RNA-seq Data

For the detection of differentially rhythmic isoforms in the RNA-seq data, the robustDODR method from the DODR R package version 0.99.2 [26] was applied to calculate the *p*-value of differential rhythmicity for each pair of rhythmic transcripts. For the baboon data, two rhythmic transcripts were defined as a pair if they had the same gene identifier and the same period in the same tissue and if one transcript had a RAIN *p* < 0.005 and the other a RAIN *p* < 0.05. For the CRC cell line data, all transcripts pairs with a RAIN *p* < 0.05 were considered. The DODR *p*-values of the total number of 12-h and 24-h rhythmic isoforms pairs per tissue were FDR-corrected using the Benjamini-Hochberg procedure and significance for differential rhythmicity was bounded by DODR *q* < 0.05. To specifically identify isoforms with similar amplitudes and a strong phase difference, only differentially rhythmic isoform pairs with a relative amplitude difference < 2 and a phase difference ≥ 2 h (for baboon tissues) or ≥ 3 h (for human cell lines) for 12-h rhythmic transcripts and ≥ 4 h for 24-h rhythmic transcripts (for both baboon and human cell lines) were retained for subsequent analyses.

### 4.9. Gene Enrichment Analysis

GO enrichment analysis (biological process—BP) of gene sets was performed using the clusterProfiler R package version 3.10.1 [84]. For the sets of murine candidate genes with rhythmic FIRMAGeneScores, org.Mm.eg.db version 3.7.0 was used for annotation and *p*-values were FDR-corrected using the Benjamini-Hochberg procedure with significance bounded by *q* < 0.05. The baboon candidate genes with differentially rhythmic phase-shifted isoform pairs were mapped to orthologous human genes using Ensembl BioMart (Ensembl Genes 95). org.Hs.eg.db version 3.7.0 was used for annotation and *p*-values were FDR-corrected using the Benjamini-Hochberg procedure with significance bounded by *q* < 0.05 for genes with 24-h rhythmic and with *p* < 0.05 for genes with 12-h rhythmic isoforms for baboon and by *p* < 0.05 for genes with 24-h rhythmic isoforms in SW480 and with *q* < 0.05 for genes with 24-h rhythmic isoforms in SW620 cells. For visualisation, GO terms were manually curated to remove redundant categories. The complete lists of results can be found in Table S4.

## Supplementary Materials

Supplementary Materials can be found at https://www.mdpi.com/1422-0067/20/16/3977/s1.
