# Supplementary material for: A Computational Analysis of Alternative Splicing across Mammalian Tissues Reveals Circadian and Ultradian Rhythms in Splicing Events
Source: Int J Mol Sci. 2019 Aug 15;20(16):3977. doi: 10.3390/ijms20163977 (PMC6721216; doi:10.3390/ijms20163977)
Supplement: Supplementary file 1 [file ijms-20-03977-s001.zip › ijms-558358 supplementary/ijms-558358-supplementary_20190816.pdf]

Supplementary Materials: A computational analysis of alternative splicing across mammalian tissues reveals circadian and ultradian rhythms in splicing events

Rukeia El-Athman[1,2], Dora Knezevic[1,2], Luise Fuhr[1,2] and Angela Relógio

Table S1. Tissues and tissue abbreviations in the baboon, mouse and human CRC cell lines datasets.

Table S2. Lists of 426 splicing-related genes in human and orthologous genes in mouse and baboon.

Table S3. 24-h and 12-h rhythmic FIRMAGene scores in murine tissues.

Table S4. Lists of enriched GO terms for genes with candidate 24-h and 12-h rhythmic AS events.

Table S5. Phase-shifted differentially 24-h and 12-h rhythmic isoform pairs in baboon tissues.

Table S6. Phase-shifted differentially 24-h and 12-h rhythmic isoform pairs in human CRC cell lines SW480 and SW620.

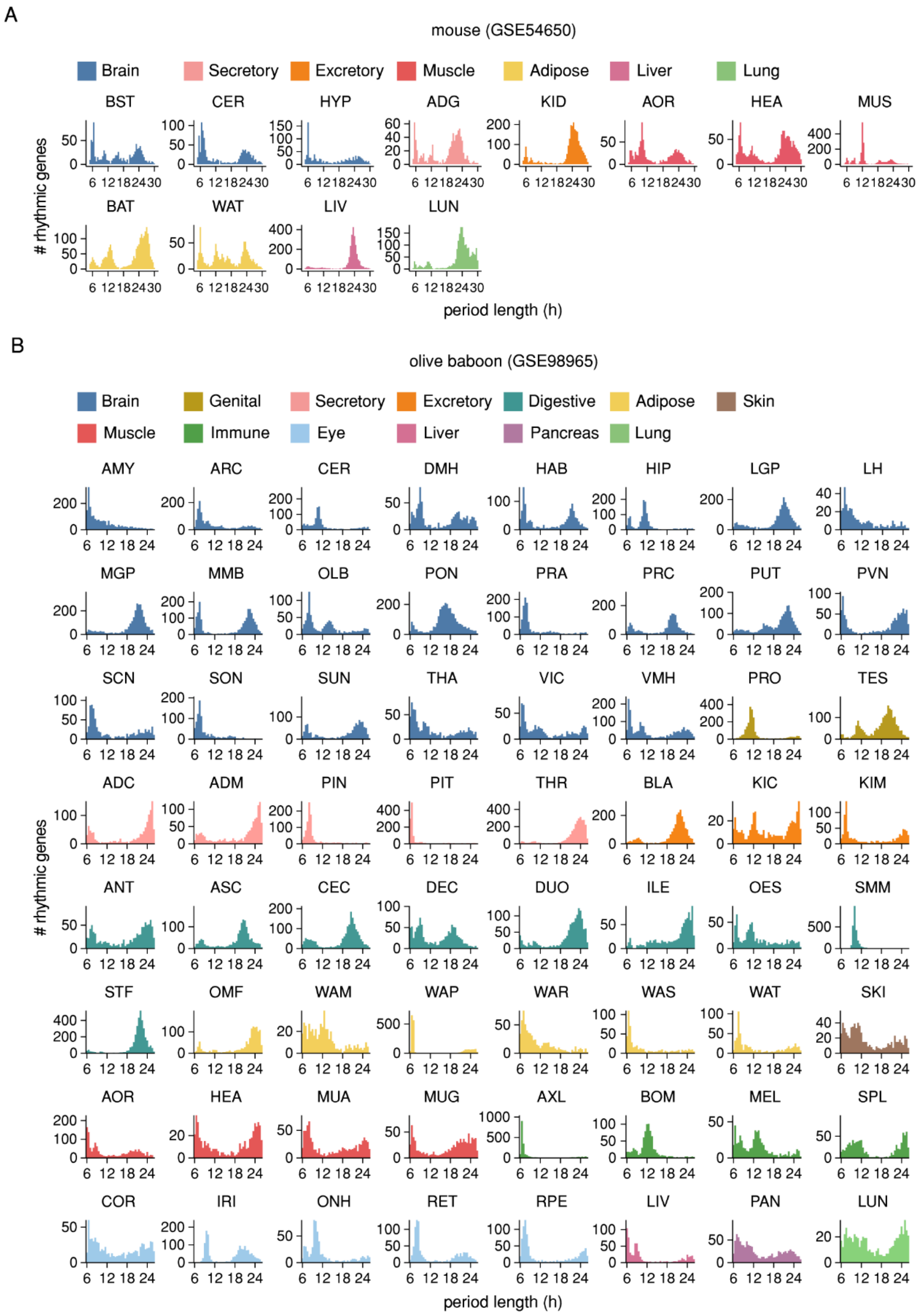

Figure 1. Period length distribution of rhythmic genes in mouse and baboon tissues. (A) Histograms of the period length distributions of rhythmic genes in twelve murine tissues. Rhythmic genes were determined based on RMA-preprocessed microarray intensity values for a period range of 5–30 h and increments of 0.1 h by fitting a harmonic regression curve ($p < 0.01$). For each rhythmic gene, only the period length with the lowest p-value is shown. Period lengths at the extreme ends of the range (5 h and 30 h) were excluded. (B) Histograms of the period length distributions of rhythmic genes in 64 baboon tissues. Rhythmic genes were determined based on CPM values for a period range of 6–26 h and increments of 0.1 h by fitting a harmonic regression curve ($p < 0.01$). For each gene, only the period length with the lowest p-value is shown. Period lengths at the extreme ends of the range (6 h and 26 h) were excluded.

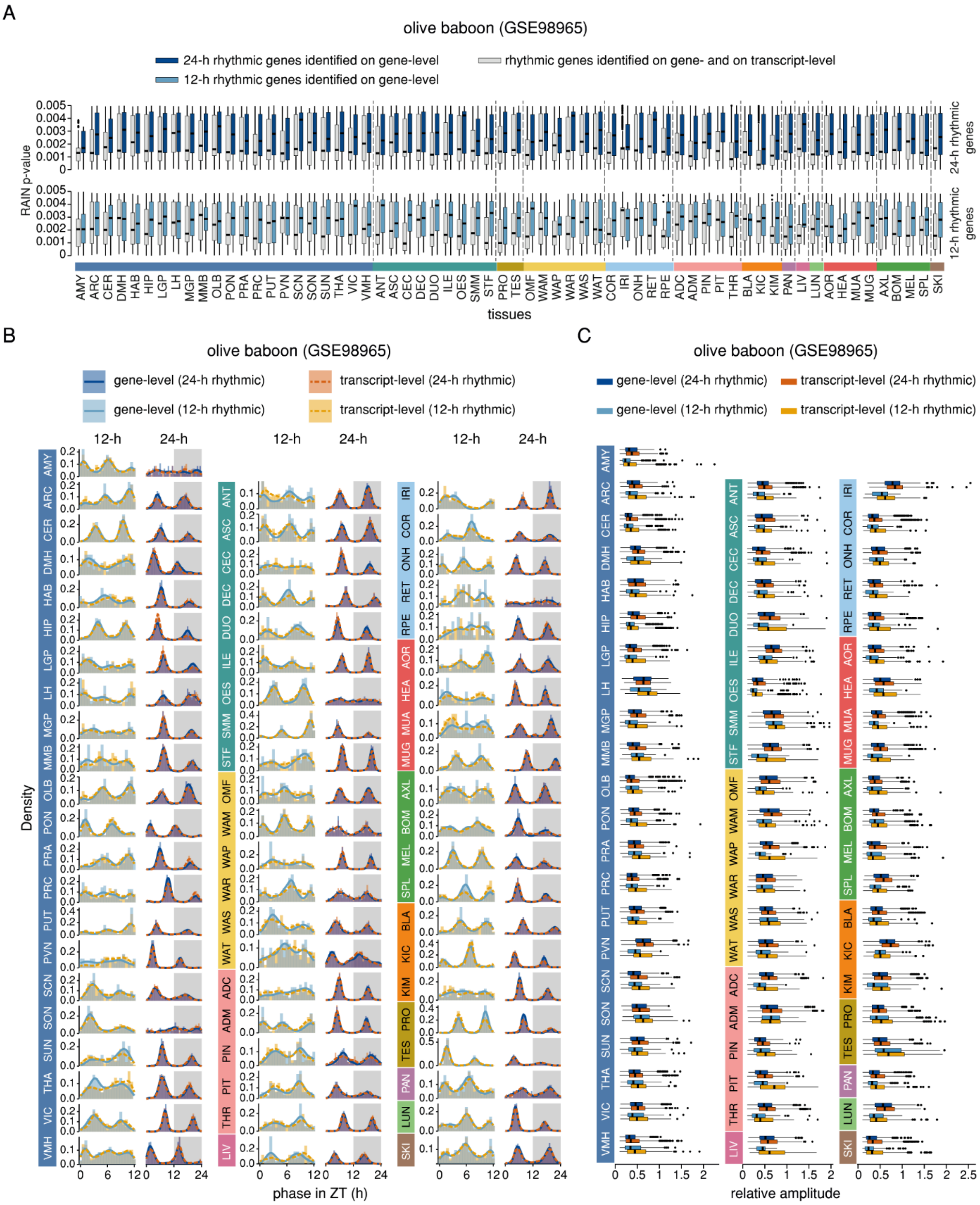

Figure 2. Comparison of rhythmic parameters between gene- and transcript-level in baboon tissues. (A) Boxplots comparing the RAIN *p*-value distributions of genes from the reverse complement (24-h rhythmic: dark blue; 12-h rhythmic: light blue) and from the intersection (light grey) of 24-h rhythmic genes on gene- and transcript-level (left panel) and the intersection (light grey) of 12-h rhythmic genes on gene- and transcript-level (right panel) identified for each baboon tissue. (B) Distribution of peak phases of 24-h rhythmic genes (dark blue) and transcripts (dark orange) and 12-h rhythmic genes (light blue) and transcripts (light orange) of all baboon tissues. (C) Boxplots of the relative amplitudes of 24-h rhythmic genes (dark blue) and transcripts (dark orange) and 12-h rhythmic genes (light blue) and transcripts (light orange) of all baboon tissues.

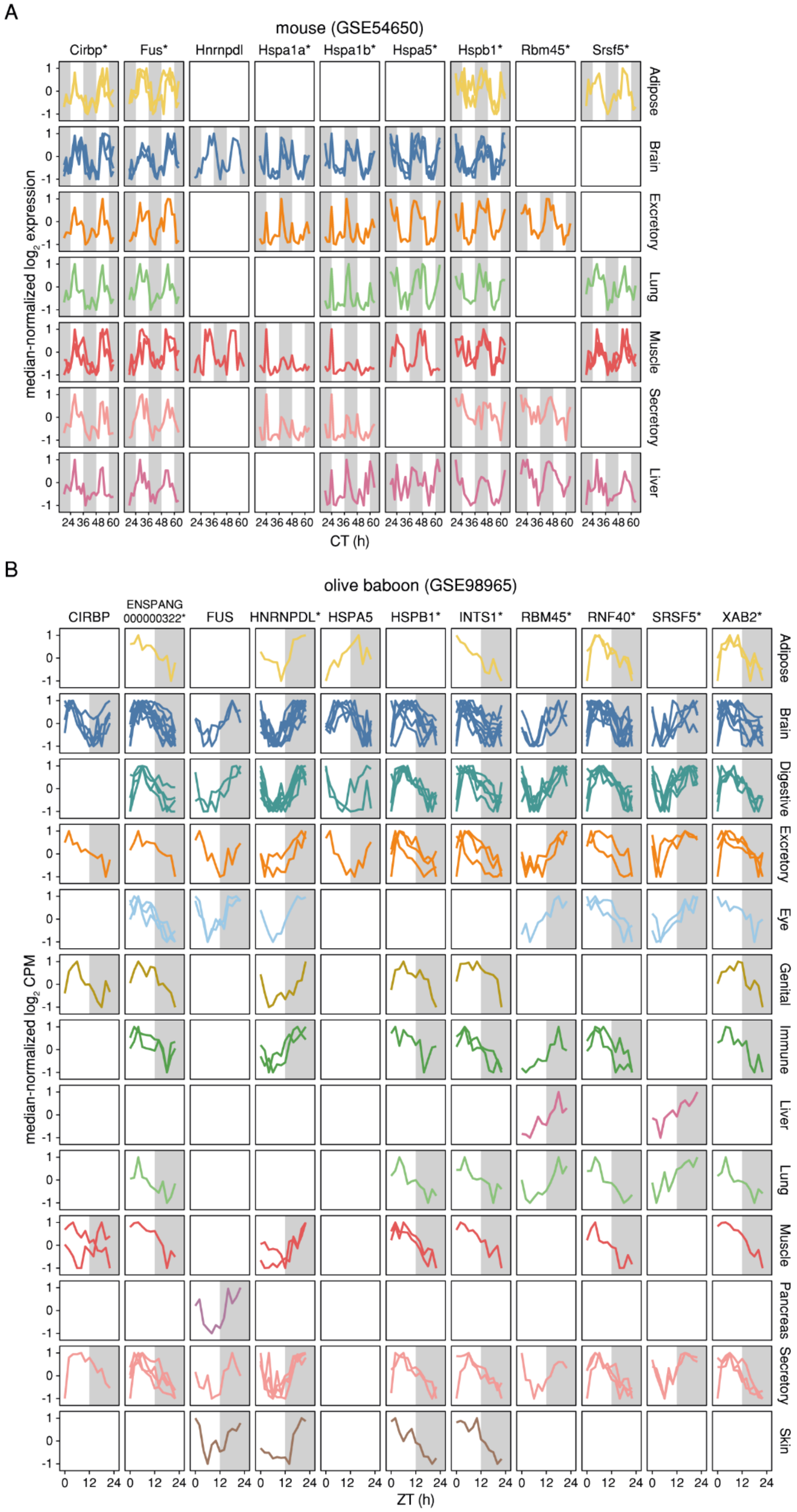

Figure 3. Expression patterns of 24-h rhythmic splicing-related genes in mouse and baboon. (A) Median-normalized expression (from -1 to 1) of the topmost five consistently 24-h cycling splicing-related genes in

murine tissues and splicing-related orthologous genes that were found to be 24-h rhythmic in both murine and baboon tissues (marked with an asterisk). (B) Median-normalized expression (from -1 to 1) of the topmost five consistently 24-h cycling splicing-related genes in baboon tissues and splicing-related orthologous genes that were found to be 24-h rhythmic in both murine and baboon tissues (marked with an asterisk). Expression is only shown for tissues in which the specific gene was found to be 24-h rhythmic. Gene expression for tissues from the same tissue type is represented with the same color and in the same panel.

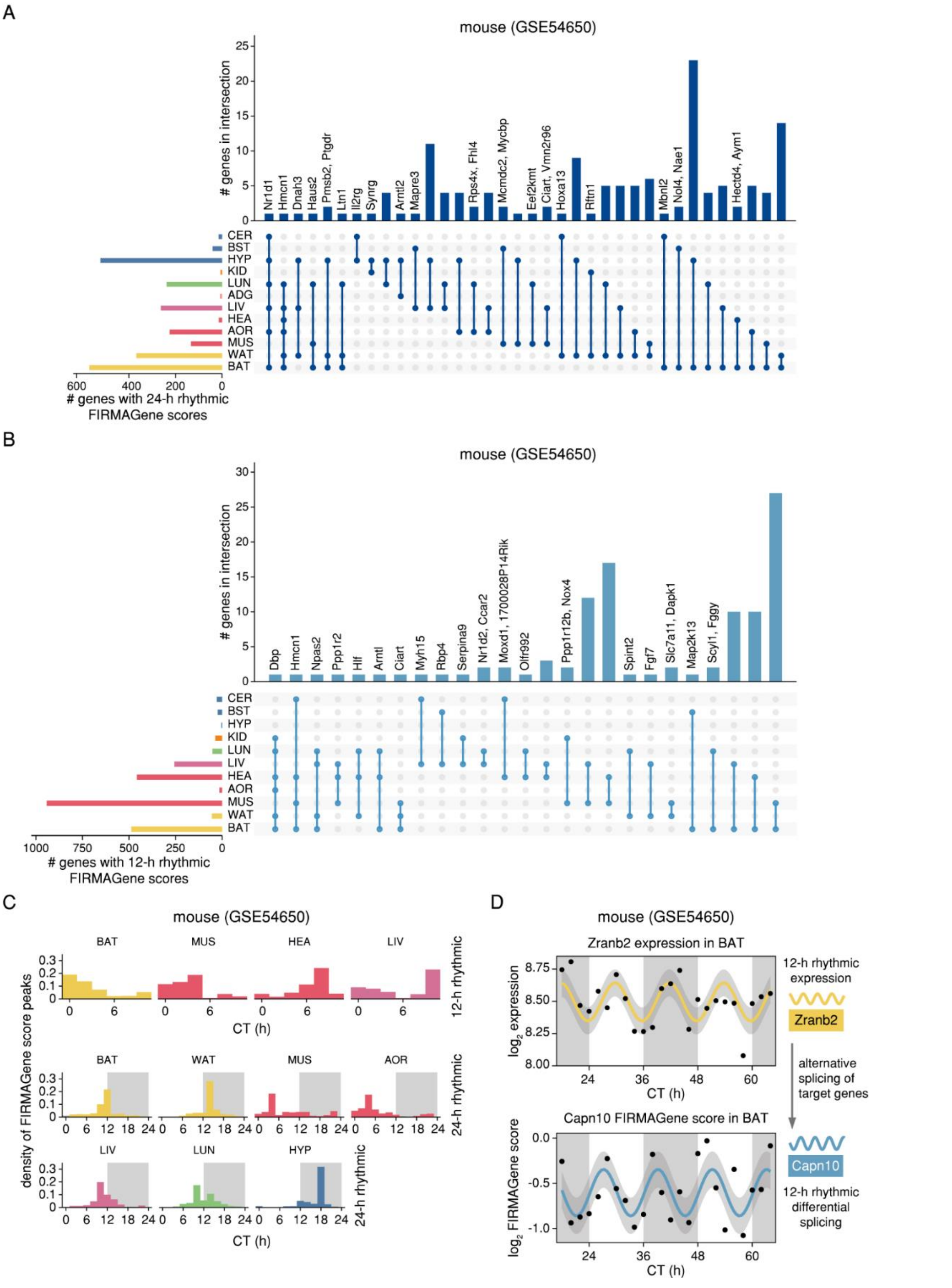

Figure 4. Candidate 24-h and 12-h rhythmic AS events in murine tissues. UpsetR plots to visualize the intersections between tissues for genes with 24-h rhythmic (A) and 12-h rhythmic (B) FIRMAGenes scores in the murine microarray dataset. (C) Distribution of the peak times of 12-h and 24-h rhythmic FIRMAGene scores for tissues in which at least 100 genes with rhythmic candidate AS events were found. (D) Expression of the gene Zranb2 and FIRMAGene score of its target gene Capn10 in murine brown fat (BAT).

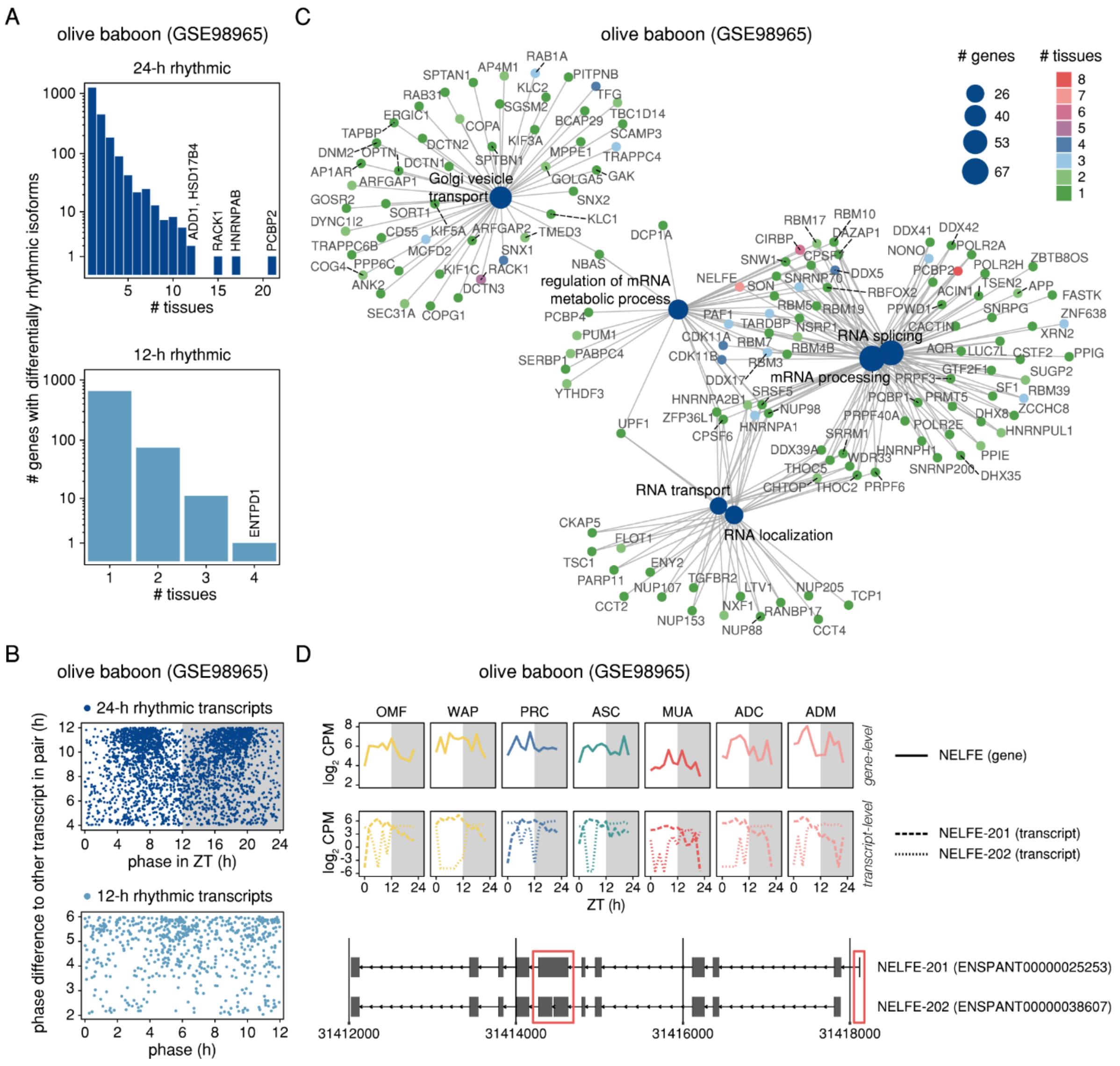

Figure 5. Candidate 24-h and 12-h rhythmic AS events in baboon tissues. (A) Distribution of the number of genes with differentially 24-h rhythmic (upper panel, dark blue) and 12-h rhythmic (lower panel, light blue) isoform pairs ranked according to the number of olive baboon tissues in which their transcripts were identified to be differentially rhythmic (DODR $q < 0.05$). (B) Phase distribution and pairwise phase-difference of phase-shifted differentially 24-h rhythmic (upper panel, dark blue) and 12-h rhythmic (lower panel, light blue) isoform pairs for all olive baboon tissues. (C) Gene-concept network representing the linkages of genes with phase-shifted differentially 24-h rhythmic isoforms pairs in olive baboon tissues and enriched GO terms (Biological Process – BP). GO term sizes are represented according to the number of linked genes. Genes are colored according to the number of tissues in which their transcripts were identified to be phase-shifted. (D) Expression (log_2 CPM) of the candidate gene NELFE (upper panel) and its transcripts NELFE-201 and NELFE-202 (middle panel) in the seven olive baboon tissues in which they were identified to be differentially 24-h rhythmic and phase-shifted and visualization of the genomic structure of NELFE transcripts (lower panel). Differences in the exonic composition are marked with red rectangles.

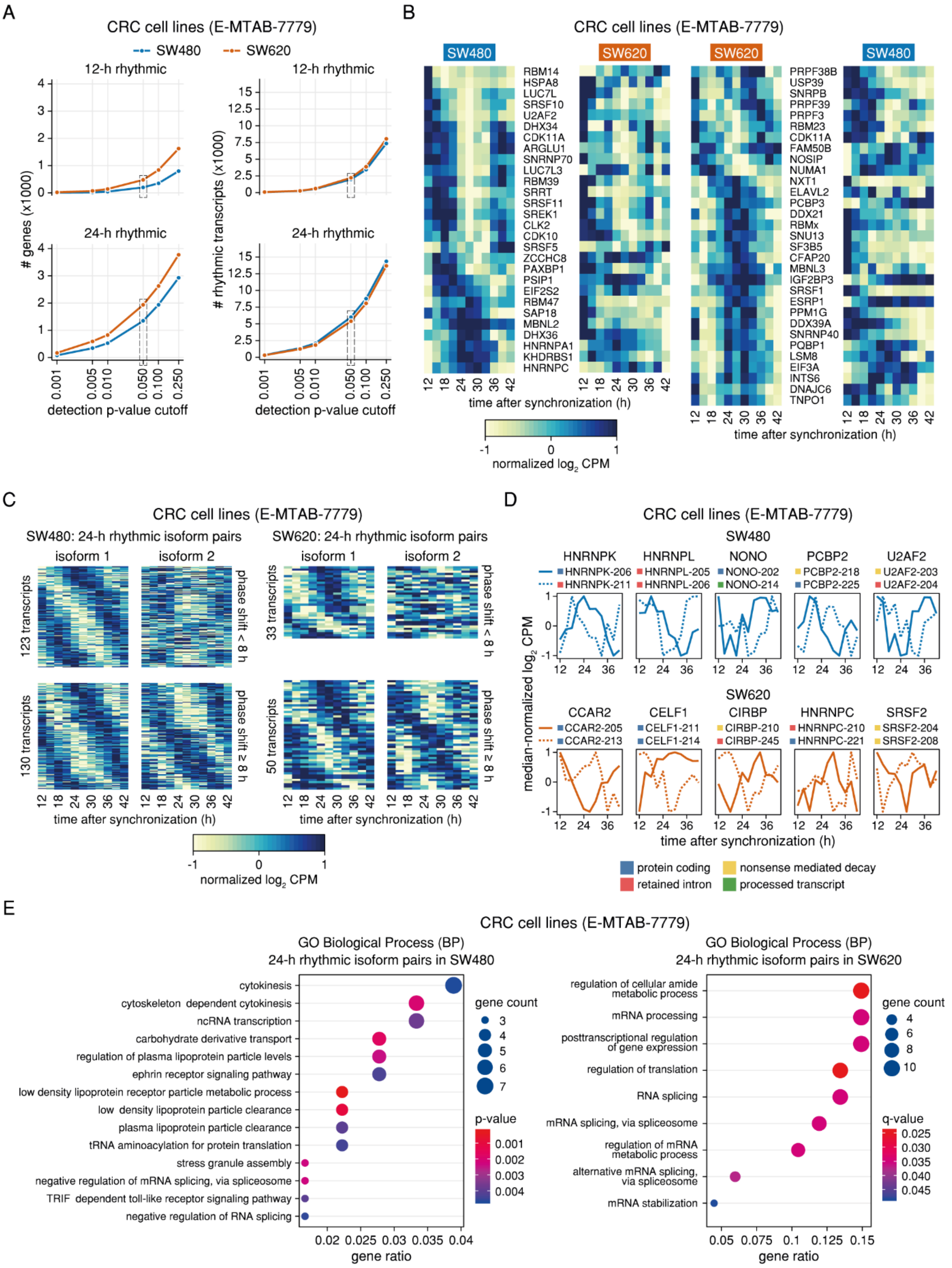

Figure S6. Rhythmicity analysis and candidate 24-h rhythmic AS events in human CRC cell lines. (A) Number of 12-h rhythmic genes (left upper panel) and transcripts (right upper panel) and 24-h rhythmic genes (left lower panel) and transcripts (right lower panel) in SW480 (blue) and SW620 (orange) for different RAIN p-value cutoff levels. (B) Phase-sorted expression heatmaps of 24-h rhythmic splicing-related genes in SW480 and their expression in SW620 (left panel) and of 24-h rhythmic splicing-related genes in SW620 and their expression in SW480. (C) Phase-sorted expression heatmaps of phase-shifted isoform pairs in SW480 (left

panel) and SW620 (right panel). Each row represents one isoform pair sorted according to the phases of isoform 1. The heatmaps are further subdivided according to the length of the phase shift between the transcripts of one isoform pair. (D) Median-normalized expression (from -1 to 1) of 24-h rhythmic phase-shifted transcript from selected splicing-related genes in SW480 (blue) and SW620 (orange). (E) Enriched GO terms (Biological Process – BP) for the sets of genes that exhibited 24-h rhythmic phase-shifted isoform pairs in SW480 (left panel) and in SW629 (right panel). For visualization, GO terms were manually curated to remove redundant categories. The complete lists of results can be found in **Table S4**.
